# Getting Ahead While Getting Along: Followership as a Key Ingredient for Shared Leadership and Reducing Team Conflict

**DOI:** 10.3389/fpsyg.2022.923150

**Published:** 2022-06-27

**Authors:** Noelle Baird, Alex J. Benson

**Affiliations:** Department of Psychology, Western University, London, ON, Canada

**Keywords:** followership, shared leadership, team conflict, social relations model (SRM), multilevel structural equation model (MSEM), leader–follower dynamics

## Abstract

Followership and leadership provide two distinct but complementary sets of behaviors that jointly contribute to positive team dynamics. Yet, followership is rarely measured in shared leadership research. Using a prospective design with a sample of leaderless project teams, we examined the interdependence of leadership and followership and how these leader-follower dynamics relate to relationship conflict at the dyadic and team level. Supporting the reciprocity of leader-follower dynamics, social relations analyses revealed that uniquely rating a teammate higher on effective leadership was associated with being rated higher by that same person on effective followership. Additionally, team members with a reputation as an effective leader also tended to be viewed as an effective follower. As expected, team levels of leadership were tightly linked to team levels of followership. Connecting these results to relationship conflict at the dyadic level, we found that uniquely rating someone as an effective follower or an effective leader would decrease the likelihood of experiencing interpersonal conflict with that person and that having a reputation for effective followership or effective leadership relates negatively to being viewed as a conflict hub within the team. Finally, effective followership was significantly negatively related to team levels of conflict, but we did not find a significant relation between effective leadership and relationship conflict at the team level. Our results highlight that followership is not only a necessary ingredient for high levels of shared leadership to exist within a team, but it underpins more functional team interactions.

## Introduction

Throughout history, audiences have been delighted by stories of the extraordinary efforts of a single leader. This lionization of one charismatic leader remains pervasive today and is reflected in the sheer volume of organizational research examining the positive impact of leaders on performance (e.g., Ceri-Booms et al., 2017). Although the fascination with individual leadership is unlikely to fade away, there has been an increasing trend toward shared leadership, a group phenomenon that describes the dynamic sharing of the leadership role within a team. In theory, shared leadership emerges because the increasingly complex demands of work groups may be too great for a single team leader to handle alone. If work is distributed among several leaders, teams should be able to generate more innovative work by leveraging the strengths of each team member.

Shared leadership is nearly universally touted as a positive and desirable team process (cf. Locke, 2003), and research asserts that having multiple sources of influence within the team benefits team performance (e.g., Carson et al., 2007; Drescher et al., 2014). At the same time, however, other lines of inquiry show how having multiple members of a team exerting their influence over one another can be detrimental to team functioning. This is supported by studies demonstrating that team functioning may suffer if too many high-status members compete for influence (e.g., Groysberg et al., 2011; Bendersky and Hays, 2012; Swaab et al., 2014). It follows that teams with many members engaging in influence behaviors may experience conflict if team members are unwilling to at times defer to other members and engage in followership behaviors. To illustrate the potentially chaotic outcome of too many teammates vying for influence in a team, consider the devastating defeat of the Miami Heat at the hands of the Dallas Mavericks in the 2011 NBA championship finals. The Heat, led by LeBron James, Dwayne Wade, and Chris Bosh (aka. “The Big Three”), could not successfully coordinate their efforts and ultimately lost the championship. The loss left many basketball fans aghast, wondering how a team with so many superstar players gracing their roster could fail to claim the title. Following the loss, one sports reporter had this to say, “The Mavericks were able to defy all odds, and prove a team always stands stronger than three stars,” (Sunnergren, 2014). This example demonstrates that simply having talented people to perform each role on a team is not enough and building a team around individual efforts cannot succeed if it is not meshed with sophisticated team coordination and cooperation.

Although shared leadership theory acknowledges the notion of dynamically “stepping up and stepping back” as crucial to the success of shared leadership (e.g., Pearce and Conger, 2003; Klein et al., 2006; Carson et al., 2007; Aime et al., 2014), empirical investigations tend to focus on expressions of effective leadership only. This captures the degree to which team members step up and influence others on the team but does not directly measure team members’ willingness to step back and defer to that person. We assert that team members’ willingness to allow others in the group to lead (i.e., followership) is a crucial, yet rarely measured, factor that enables processes such as shared leadership to result in positive team outcomes.

In the current study, we propose that followership is a necessary component of the shared leadership model and investigate the dyadic interplay between leadership and followership in leaderless project teams (i.e., teams without a formally appointed leader). The second objective of our research is to examine the unique variance accounted for by both followership and leadership in predicting both dyadic and team conflict states. Specifically, we investigate how impressions of team members’ leadership and followership effectiveness relate to subsequent perceptions of conflict between team members as well as conflict at the team level.

By measuring leadership and followership in tandem and examining their influence on subsequent team conflict states, we make three contributions. First, we fill an important gap in the shared leadership literature by zooming in on the dyadic underpinnings of leader-follower dynamics in teams. Shared leadership is a team level construct that is typically formed by aggregating leadership ratings (e.g., Wang et al., 2014) or examining the distribution of leadership throughout the team with network analysis (e.g., Small and Rentsch, 2011). Our study aims to unpack the “black box” of shared leadership by examining how bottom-up processes (i.e., interpersonal dynamics) enable shared leadership structures to flourish in teams. Using social relations modeling (SRM; e.g., Back and Kenny, 2010), we provide insight into how leadership and followership behaviors covary in teams. This approach answers recent calls by Zhu et al. (2018) to leverage new methods that precisely capture important aspects of shared leadership. Second, we also use SRM to examine how team members’ ratings of each other’s leadership and followership behaviors affect the emergence of relationship conflict between team members. Past research has shown that teams with shared leadership (operationalized as the number of members engaging in leadership behaviors) tend to experience less conflict (Bergman et al., 2012). However, it has been suggested that team dynamics can be best understood as the cumulative expression of interaction patterns between team members (Humphrey and Aime, 2014), necessitating the examination of the dyadic origins of team phenomena. We extend this line of inquiry by examining how expressions of effective leadership and followership affect the emergence of relationship conflict within dyads and how these may contribute to a reputation for conflict throughout the team. Finally, although team conflict is a well-studied phenomenon, we are unaware of any studies that have examined how dyadic and shared perceptions of *both* effective leadership and followership may potentially buffer against the emergence of relationship conflict. As relationship conflict can be detrimental to team performance and satisfaction (e.g., De Dreu and Weingart, 2003), the results of this study could have important implications for teams wishing to keep their conflict levels at a minimum.

## Hypothesis Development

### Shared Leadership and the Interdependence of Leadership and Followership

This study synthesizes theory pertaining to shared leadership in teams with the leadership process model (Uhl-Bien et al., 2014) to predict team conflict outcomes. Shared leadership draws on concepts from both role theory and social exchange theory. Role theory (e.g., Katz and Kahn, 1978) describes the progressively meaningful evolution of interconnected roles as individuals interact with one another over time. The more individuals within a work unit engage with one another, the more they begin to understand each others’ roles, how they fit together, and their place in the larger collective (e.g., a team). Over time, this process of role sense-making becomes the foundation for group norms and expectations regarding how team members should act in relation to one another. When the result of this process is a balanced pattern of reciprocal influence among team members, this is said to be the foundation of shared leadership (Seers et al., 2003).

The concept of reciprocity is also central in social exchange theory. According to the theory, exchange relationships involve reciprocal interdependence, whereby the actions of one party elicit a complementary response from the other party (Molm, 1994). In the case of shared leadership, this applies to enacting influence attempts and allowing oneself to be influenced. Further, the notion of reciprocity implies that in interdependent exchange relationships, there is an expectation that the other party will respond in kind at a later date (Seers et al., 2003). Indeed, it has been noted that, at the dyadic level, “influence over others is purchased at the price of allowing oneself to be influenced by others” (Homans, 1961, p. 286). This continuous, reciprocal act of influencing others and allowing oneself to be influenced is then extended to the group level in theories of shared leadership. Group exchange relationships operate in a similar way; however, it is now up to the group as a collective to decide whether influence attempts are situationally appropriate and should be reinforced with a complementary response (Seibert et al., 2003). The group’s decision to legitimize the influence attempt can be based in several factors, including positive group norms surrounding influence attempts from group members (Seers et al., 2003) or characteristics of individual group members (e.g., relevant knowledge or skills; Aime et al., 2014 or history of positive contributions to the collective; Seers et al., 2003).

Considering these insights from role and exchange theory together, shared leadership is a relational phenomenon that is rooted in group member interactions at both the dyadic and group level and characterized by the mutual exchange of influence among team members over time. These theoretical roots are well documented (see Pearce and Conger, 2003), however, the ways in which shared leadership is operationalized do not always reflect the nuances present in theoretical descriptions of the construct. A notable exception comes from Aime et al. (2014), who empirically tested the notion of dynamic power shifts in teams and demonstrated that shared leadership can indeed promote creativity in teams, so long as the person whose expertise holds the most relevance takes on the leadership role and their claim to leadership is perceived as legitimate. This work undoubtedly advances our understanding of how shared leadership works in teams, however, several research questions remain unanswered. Specifically, studies have yet to examine the fundamental interplay between leadership and followership in shared leadership structures.

It is clear from the theoretical accounts of shared leadership summarized above that the mutual exchange of influence does not work if group members are not committed to allowing themselves to be influenced. That is, acts of leadership must be met with complementary acts of followership for shared leadership to flourish. To aid in our theorizing of how leadership and followership represent distinct, interdependent, and complementary acts within a shared leadership arrangement, we draw on the leadership process model (Uhl-Bien et al., 2014). According to this model, leading and following represent patterns of behaviors that combine to produce “leadership.” The model is a useful starting point for considering how effective leading and following behaviors may work in tandem to facilitate the sharing of the leadership role throughout a team. Indeed, Uhl-Bien and Carsten (2018) stated that, “scholars currently studying shared leadership…are particularly well positioned for using the behavioral (co-creation) approach” (p. 213). This is because the leadership process model recognizes that expressions of leadership behaviors (e.g., delegating) must necessarily be met with the behavioral choice to follow (e.g., accepting the role delegated to them). As such, examining shared leadership as the rotating of leadership roles without considering how acts of followership allow for this to happen results in an incomplete understanding of the shared leadership construct (Shamir, 2012).

**FIGURE 1:**
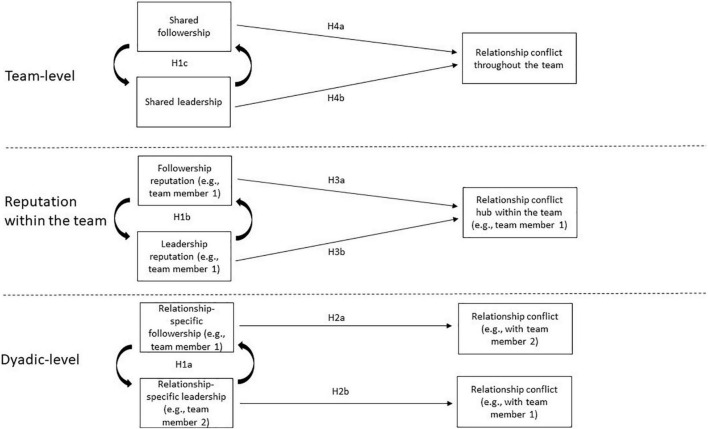
Conceptual model of main hypotheses.

To address this shortcoming, our first set of hypotheses pertain to the fundamental assumption that expressions of followership and leadership are complementary processes that unfold at the dyadic level, which provide a basis for the emergence of team level patterns of followership and leadership (see Figure 1 for our conceptual model). Theoretical accounts of shared leadership emphasize the importance of reciprocal, complementary acts of influence for the successful implementation of shared leadership (e.g., Seers et al., 2003). That is, influence attempts are met with the complementary response of allowing oneself to be influenced, with the implication that the favor may be returned in the future (e.g., Homans, 1961). Similarly, the leadership process model describes the complementary nature of leading and following behaviors, and emphasizes the need for both leadership claims and followership grants in the co-production of leadership (Uhl-Bien and Carsten, 2018). Integrating these ideas, the expression of followership toward a teammate should be tightly connected to that same teammate’s expression of leadership. As such, our first prediction emphasizes the reciprocal nature of how the expression of followership from one partner is closely linked to the expression of leadership by the other. Specifically, we hypothesize:

**Hypothesis 1a.** At the dyadic level, individuals are more likely to display effective followership toward those who they uniquely rate as a particularly effective leader.

The complementary nature of leadership and followership in dyadic interaction patterns does not imply that those who engage in followership are mutually excluded from engaging in leadership. In fact, head coaches of highly competitive sport teams described some of their strongest team leaders as those who understood when to empower others’ attempts at leadership (Benson et al., 2016). This is aligned with theoretical accounts of shared leadership (e.g., Pearce and Conger, 2003) that cite the ability to influence others and allow oneself to be influenced as fundamental to the premise of shared leadership. Moreover, in egalitarian self-managed teams, the primary context in which shared leadership is studied, there are ample opportunities for individuals to engage in both acts of leadership and followership. In such team settings, overly dominant group members may struggle to secure the support of others in their attempts to unilaterally influence the group (Redhead et al., 2019). It should also be noted that certain skills and behaviors are important for both roles (e.g., competence; teamwork skills) and thus are not unique to effective leadership or effective followership. Taken together, although leading and following represent distinct role-related behaviors, effective team members in leaderless teams are likely those who are able to flexibly navigate between the leader and follower role. As such, our second hypothesis emphasizes that the expression of followership by a team member should positively contribute to that same person’s ability to lead.

**Hypothesis 1b.** Being rated as an effective follower will relate positively to being rated as an effective leader.

Finally, as leading and following represent distinct yet complementary role-related behaviors, the degree to which team members effectively share leadership duties should be tightly linked to effective followership within the team. Because functional team dynamics in teams without a formal hierarchy theoretically rely on the willingness of teammates to engage in both leadership and followership behaviors, it is likely the case that team members who are rated as effective leaders also demonstrate the communal and cooperative tendencies present in effective followership. As such, we advance the following:

**Hypothesis 1c.** Team levels of effective followership will positively relate to team levels of effective leadership.

Not only are leading and following behaviors inextricably linked, but their co-occurrence is also necessary for effective team functioning. Further, leadership attempts that are met with non-following behaviors (e.g., resistance) or reciprocated with another leadership attempt (e.g., competition), may prohibit the effective sharing of the leadership role and cause strife within teams. Overall, a key goal of the current study is to examine how both leadership and followership buffer against dysfunctional team outcomes, namely, team conflict.

### Leadership, Followership, and Team Conflict

Dysfunctional leader–follower dynamics have the potential to derail team functioning and create a toxic social environment. Intragroup conflict is a behavioral team process that can be defined as disagreements or perceived incompatibilities within a team (e.g., De Dreu and Weingart, 2003). Three main types of team conflict have been identified in the literature: *relationship conflict*, which involves interpersonal tension between teammates (Jehn, 1997); *process conflict*, which describes “disagreements about assignments of duties and resources” (Jehn, 1997, p. 540); and *task conflict*, traditionally defined as differing opinions, ideas, or thoughts about the content of the task (Wall and Nolan, 1986; Jehn, 1995). Although task conflict can be beneficial to team performance given the right circumstances (e.g., O’Neill et al., 2018) and researchers have encouraged organizations to cultivate and embrace constructive conflict (e.g., Tjosvold, 2008), the current paper exclusively focuses on the dysfunctional side of team conflict, namely relationship conflict. Of the three types of conflict identified by Jehn (1995; 1997), relationship conflict is perhaps the most straightforward in its effects on team functioning. Simply put, interpersonal tension decreases team morale and distracts team members from the task at hand, harming productivity (Jehn, 1997). Indeed, relationship conflict has been consistently linked to lower member satisfaction and poor group performance (e.g., De Dreu and Weingart, 2003). Given the potentially detrimental effects of relationship conflict, and the interpersonal nature of its origins, this study will focus on the role of leadership and followership in relationship conflict at both the dyadic and team level.

#### Team Conflict at the Dyadic Level: Bad Apples and Sworn Enemies

Team conflict is typically examined at the collective level; however, scholars have begun to conceive of groups as a collection of *relationships* rather than an assembly of individuals (Humphrey et al., 2017). This perspective necessitates the examination of team constructs at the dyadic level to achieve a complete understanding of intrateam dynamics. Relationship conflict lends itself particularly well to a dyadic approach, as this form of conflict is already theorized to be interpersonal in nature (Jehn, 1997). Research examining the dyadic nature of relationship conflict suggests that measuring group members’ perceptions of conflict within a dyad (i.e., between themselves and another group member) and between other group members (i.e., observing conflict between other dyads) is important for understanding the negative effects of conflict in teams (e.g., Park et al., 2020). This is because perceptions of relationship conflict between oneself and another team member or between others in the team can affect whether and how team members engage in task-related behaviors with one another (Park et al., 2020). Additionally, the presence of conflict between one or more individuals in a team can encourage the formation of coalitions, further disrupting team functioning and negatively impacting relationships between team members who are not the source of the conflict (Jehn et al., 2013). Taken together, it is evident that the dyadic perspective provides a rich lens for studying the origins and outcomes of intrateam conflict. In the present study, we adopt this dyadic approach by examining team members’ perceptions of each other’s effective leadership and followership behaviors, and how these relate to subsequent dyadic relationship conflict and reputation for conflict.

According to a recent study, team conflict originates most commonly from two sources: a) two team members who cannot get along with each other (i.e., dyadic conflict) or b) one team member who instigates conflict with everyone (i.e., a “bad apple”; see Shah et al., 2021). Dyadic relationship conflict reflects personal disagreements or clashes in personality that are not associated with the task; however, research has shown that relationship conflicts can emerge when the team fails to solve task-relevant problems effectively and calmly (Yang and Mossholder, 2004). That is, disagreements around the task (i.e., task conflict) can reveal personality differences and become personal in nature. Following this logic, it is likely that perceiving a group member as failing to contribute to the successful coordination of group efforts may devolve into a personal dislike for that teammate. Effective leadership and followership are defined by behaviors that fit together to promote group functioning, with leadership focused on taking charge of solutions and followership focused on supporting and enacting those solutions (e.g., Hurwitz and Hurwitz, 2015). Further, an important feature of effective followership is cooperation (e.g., Agho, 2009), which has been identified as a potential buffer against interpersonal conflicts in teams (Weingart and Jehn, 2017). This is because cooperation involves a greater focus on the needs and feelings of others compared to oneself, thereby decreasing the likelihood of responding with insults, disrespect, etc. Although not inherently cooperative, effective leadership can promote cooperation in others by mobilizing team members to work toward a common goal or coming up with solutions for how to delegate work to maximize cooperation (Bastardoz and Van Vugt, 2019). Therefore, we assert that one’s perceptions of a group member’s effective followership and leadership behaviors will have an impact on whether you will experience conflict with this person in the future.

**Hypothesis 2a:** Uniquely rating a teammate higher in effective followership will relate to experiencing lower levels of relationship conflict with that same person.

**Hypothesis 2b:** Uniquely rating a teammate higher in effective leadership will relate to experiencing lower levels of relationship conflict with that same person.

Individuals who have been identified as a “bad apple” within the team demonstrate consistent negative behaviors that detract from optimal team functioning (e.g., withdrawing effort, having a chronic negative attitude, and acting in a way that violates group norms for appropriate conduct; Felps et al., 2006). In contrast, leadership and followership are both thought to be adaptive processes that have evolved to aid in the achievement of collective pursuits (Bastardoz and Van Vugt, 2019). As such, individuals who are rated by their team members as exhibiting behaviors that aid in the instigation or organizing of group efforts (i.e., effective leadership) and/or exhibiting behaviors that support and facilitate the goals of the team (i.e., effective followership) are unlikely to be identified as a source of conflict within the team (i.e., bad apples). Indeed, past research has found that individuals’ reputation for effective interpersonal styles, such as collaborative conflict management, reduces the likelihood of becoming both the target and the instigator of incivility (Trudel and Reio, 2011). That is, individuals who establish a reputation for collaborative and cooperative interpersonal behavior are less likely to be involved in subsequent negative (i.e., uncivil) interactions with fellow workers.

**Hypothesis 3a:** The degree to which someone has a reputation as an effective follower in the team will relate negatively to being viewed as a conflict hub within the team.

**Hypothesis 3b:** The degree to which someone has a reputation as an effective leader in the team will relate negatively to being viewed as a conflict hub within the team.

#### Connecting Dyadic Processes to the Team-Level

Interpersonal friction between pairs of individuals may negatively affect the entire group (Shah et al., 2021). For example, dyadic relationship conflict reduces the amount of information sharing throughout the team (Humphrey et al., 2017). Fortunately, the presence of high-quality exchange relationships among team members can act as a buffer against these detrimental interpersonal dynamics (i.e., TMX; de Jong et al., 2014). That is, the negative impact of being engaged in, or witnessing, conflict between teammates can be mitigated by experiencing the exchange of mutual support and information with other teammates. Following this notion that positive team functioning acts as a neutralizer to harmful interpersonal interactions, we assert that effective expressions of both leadership and followership in teams can prevent the onset of harmful team conflict states (i.e., relationship conflict) at the group level.

The contribution of effective leadership to team functioning is well documented in the literature (e.g., Ceri-Booms et al., 2017). Acts of leadership are agentic in nature, functioning to unite group members around a shared vision and mobilize members’ efforts toward collective goal attainment. A leader’s ability to create a shared vision for the team reduces conflict that is both cognitive (i.e., process and task-related) and relational in nature by prioritizing the goals of the team over self-interests (Doucet et al., 2009). Transformational leadership behaviors have also been linked to cooperative approaches to conflict management, facilitating the exchange of ideas and consideration of alternate perspectives (Zhang et al., 2011). Acts of followership, in contrast, are more communal and thereby provide the social scaffolding necessary for cooperation and coordination. Further, followers can create the conditions for optimal team functioning by modeling desirable behaviors for other followers (i.e., social facilitation; Weber and Moore, 2014). Extending this logic, followers may be able to actively reduce conflict by consistently and publicly engaging in effective followership behaviors (e.g., cooperation), thereby establishing behavioral norms for the rest of the team. Thus, followership and leadership provide two distinct yet complementary sets of role-related behaviors that jointly contribute to the mitigation of harmful group processes such as interpersonal conflict. Taken together, the research summarized above supports the notion that effective leadership and followership are both required to achieve optimal team dynamics, which provided a basis for the following hypotheses:

**Hypothesis 4a.** High team levels of effective followership will negatively relate to subsequent levels of relationship conflict.

**Hypothesis 4b.** High team levels of effective leadership will negatively relate to subsequent levels of relationship conflict.

## Methods

### Participants and Procedure

Participants were engineering students at a large Canadian university who were enrolled in a project design course and worked together in project teams from September 2018 to March 2019. Participants worked together interdependently on a team project that lasted the duration of their team’s lifecycle. Participants were assigned to their teams at the beginning of the academic year (i.e., early September)—at which point demographic data was collected. The sample consisted of 429 participants organized into 104 teams, although we were only able to extract target effects (detailed in the analysis section) for 405 participants from 98 teams. The average age was 18.13 years old (*SD* = 1.45), and the sample was 76.46% male. Given that women are underrepresented in engineering, the course instructor requested that teams were composed exclusively of men (51.38% of teams) or had at least two women (48.62% of the teams were mixed-gender). Institutional ethics approval was obtained prior to conducting the study. Participants spent several months interacting and getting to know each other before providing their ratings of their teammates’ leadership and followership behaviors. Data were collected online in the second academic term; three weeks into January 2019 and two weeks into March 2019, with seven weeks between the two measurement periods^1^.

### Measures

To facilitate dyad level analyses, participants were instructed to rate every member of their group, even those who were absent that day. Participants were informed that their responses on this item would not be seen by their teammates or the course instructor, and the ratings given would have no bearing on their final grades. For all round-robin measures, we decomposed the variance of peer ratings into target, perceiver, and relationship components using SRM.

#### Effective Followership (Time Point 1)

Effective followership was assessed using the relative percentile method (RPM). Informed by theory on the nature of followership (Uhl-Bien et al., 2014) and leaders’ descriptions of effective followership in teams (Benson et al., 2016), participants were provided a definition of effective followership (i.e., “Effective followers help to support others’ decisions, put team goals ahead of self-interests, and carry-out the tasks needed to accomplish team goals”), and then asked to rate the extent to which each teammate demonstrates effective followership on a 101-point sliding scale. The RPM has higher criterion-related validity and greater accuracy than absolute rating methods of performance, and is especially effective in cases where multiple raters are providing scores for the same rate (Goffin and Olson, 2011).

#### Effective Leadership (Time Point 1)

Effective leadership was also assessed using the RPM on a 101-point sliding scale. Participants were shown a definition of effective leadership that was derived from leadership research (e.g., Yukl, 2012; “Delegate tasks to others, show initiative, motivate team members, and unite members in accomplishing team goals”) and subsequently rated the degree to which each teammate engaged in effective leadership behaviors relative to all other teammates the respondent had ever worked with.

#### Dyadic Relationship Conflict (Time Point 2)

Participants rated their relationship conflict with each team member on a scale from 1 (*none at all*) to 5 (*all the time*). Relationship conflict was measured using a single item in a round-robin format [“How often did you engage in negative conflict (e.g., conflict regarding relationships, personal attacks or status) with other members of your team?”].

#### Team-Referent Relationship Conflict (Time Point 2)

Relationship conflict at the team level was measured using Behfar et al.’s (2011) team-referent measure. Responses on this scale range from 1 (*a very small amount*) to 5 (*a lot*). The relationship conflict subscale contained four items assessing the interpersonal problems that may be happening within the team (e.g., “How much friction is there among members of your team?”).

### Analytic Procedure

#### Dyadic Analyses

We used SRM to test the dyadic interplay between followership, leadership, and relationship conflict. Although shared followership and leadership are team level phenomena, Humphrey and Aime (2014) conceptualized team dynamics as the cumulative expression of dyadic interaction patterns. As such, we used SRM as a vehicle to zoom in on how teammates rate each other on their leadership, followership, and conflict. SRM recognizes that in each of these ratings, three main components affect the scores: the tendencies of the rater (i.e., the *perceiver* effect), the reputation of the ratee (i.e., the *target* effect), and the unique relationship between the two people (i.e., the *relationship* effect). For the purposes of this study, we used the TripleR v. 1.5.3 package with the multiple group option for R (Schönbrodt et al., 2012) to first decompose the peer ratings of leadership, followership, and relationship conflict into these component parts. This provided a basis for the subsequent dyadic analyses.

First, we explored the interdependence of followership and leadership at the dyadic level. Using bivariate social relation analyses, we assessed the interplay between followership and leadership by examining (a) how much uniquely rating a teammate higher (or lower) on followership relates to being rated higher (or lower) by that same person on leadership (i.e., interpersonal relationship correlation, Hypothesis 1a), (b) how much uniquely rating another person as a leader relates to rating that same person as a follower (the intrapersonal relationship correlation), and (c) the degree to which someone has a reputation as an effective (or ineffective) follower in the team is related to being viewed as an effective (or ineffective) leader (target-target covariance between followership and leadership, Hypothesis 1b).

The next set of analyses aimed to unpack how these followership and leadership processes connects to relationship conflict at the dyadic level. First, we calculated the dyadic reciprocity index to evaluate the assumption that a perceiver’s rating of relationship conflict is linked to a target’s perception of relationship conflict between pairs of teammates. Second, we conducted bivariate analyses focused on time-lagged associations pertaining to (a) how much uniquely rating a teammate lower in followership effectiveness is linked to experiencing relationship conflict with that same person (i.e., intrapersonal relationship correlation, Hypothesis 2a) and (b) the degree to which someone has a reputation as an effective (or ineffective) follower in the team is related to being viewed as a conflict hub within the team (target-target covariance between followership and relationship conflict, Hypothesis 3a). Following this same approach, we assessed time-lagged associations pertaining to how effective leadership is associated with relationship conflict via the intrapersonal relationship correlation (Hypothesis 2b) and target-target covariance (Hypothesis 3b).

#### Connecting Dyadic Processes to the Team-Level

Finally, we used the extracted target effects of followership, leadership, and relationship conflict, as well as a team-referent measure of relationship conflict, to examine the nature of these relations at the team level (Hypotheses 1c, 4a, and 4b). Specifically, we regressed our dyad-referent and team-referent measures of relationship conflict onto the extracted target effects of followership and leadership. Due to the hierarchical nature of the data (i.e., individuals nested within teams), multilevel structural equation modeling was used to partition within-team variance from between-team variance via latent decomposition, and test these associations (Lüdtke et al., 2008; Muthén and Muthén, 2017). That is, latent team-level scores were estimated for followership, leadership, and relationship conflict, and these were orthogonal to the within-team estimates for each variable. Studies have shown that traditional measures of leadership can successfully be aggregated and translated to the team level (e.g., Avolio et al., 2003). We used maximum likelihood estimation for missing data with a sandwich estimator that produces standard errors robust to non-normality (i.e., MLR, Mplus version 8.2; Muthén and Muthén, 2017). With regards to missing data, the analysis used peer reports where respondents were instructed to rate all teammates even those who were not in attendance at the data collection session. Therefore, even missing team members would have a score on the peer-rated measure of effective leadership and followership. However, extracting target effects for followership, leadership, and dyad-referent relationship conflict via SRM required at least two members reporting on each individual within a given team. As such, teams with missing members were retained in the analysis, provided that the team had more than one member in attendance. As noted by O’Neill et al. (2018), teams with low attendance could be teams that are experiencing intrateam conflict and deleting such teams may lead to biased estimates due to including only the high functioning teams. We did, however, exclude participants who changed teams between the first point of measurement and the second to ensure that participants were providing team conflict ratings for the same team members whose leadership and followership they were rating earlier in the term in the lagged analysis. We ran two separate models with effective leadership and followership, both at the team and individual level, as the predictors and each relationship conflict measure as the criterion variable (i.e., dyad-referent and team-referent measures). Our useable sample was 380 participants from 98 teams for the analyses predicting group-referent relationship conflict, and 332 participants from 81 teams for the analyses predicting dyad-referent relationship conflict.

## Results

Table 1 contains the descriptive statistics and intercorrelations between the variables.

**TABLE 1:** Descriptive statistics and intercorrelations between the variables.

Variable	1	2	3	4
(1) Followership^a^	−	0.77***	–0.39***	–0.36***
(2) Leadership^a^	0.35***	–	–0.10	–0.14
(3) Dyad-referent Relationship Conflict^b^	−0.44***	–0.19*	–	0.70***
(4) Team-referent Relationship Conflict^a^	0.04	–0.02	–0.01	–
*M*	77.79	71.56	1.34	1.80
*SD*	8.71	10.12	0.40	0.67

*Numbers below the diagonal refers to observed cluster-mean centered scores at the individual-level (n^a^ = 405; n^b^ = 385), whereas numbers above the diagonal refers to observed mean scores at the team-level (k = 98).*

*M = mean at the team-level; SD = standard deviation at the team-level.*

*p < 0.05; **p < 0.01; ***p < 0.01.

### Dyadic Interplay of Followership, Leadership, and Relationship Conflict

Specific to Hypothesis 1a, the level of followership effectiveness exhibited by one interaction partner was tightly connected to the leadership effectiveness of the other interaction partner at the dyadic level. Specifically, the positive interpersonal relationship covariance showed that uniquely rating a teammate higher on followership was associated with being rated higher by that same person on leadership (*b* = 13.28, *SE* = 5.64, *t* = 2.35, *p* = 0.010). The positive intrapersonal relationship covariance indicated that perceivers who uniquely rated a teammate higher in leadership tended to rate that same teammate higher in followership (*b* = 35.27, *SE* = 5.64, *t* = 6.25, *p* < 0.001). Beyond dyad-specific effects, we also investigated individuals’ reputation within the team (i.e., target effects). In line with Hypothesis 1b, target effects of followership positively correlated with target effects of leadership (*b* = 35.30, *SE* = 9.91, *t* = 3.56, *p* < 0.001)^2^.

The dyadic reciprocity for relationship conflict revealed that between pairs of teammates, uniquely perceiving conflict with a given teammate tended to be reciprocated by that target (*b* = 0.04, *SE* = 0.01, *t* = 2.84, *p* = 0.005)^3^. Continuing the emphasis on dyad-level relations, we then evaluated the extent to which followership and leadership effectiveness at time point one were associated with relationship conflict at time point two. Supporting Hypothesis 2a, the positive intrapersonal relationship covariance demonstrated that perceivers who uniquely rated a specific teammate higher in followership tended to experience less relationship conflict with that teammate (*b* = –0.72, *SE* = 0.25, *t* = –2.85, *p* = 0.002). Likewise, in line with Hypothesis 3a, individuals’ reputations for followership effectiveness within the team (i.e., target effects) were negatively correlated with target effects of relationship conflict (*b* = –1.17, *SE* = 0.33, *t* = –3.55, *p* < 0.001). We observed a similar pattern for leadership effectiveness and relationship conflict. Supporting Hypothesis 2b, perceivers who uniquely rated a specific teammate higher in leadership tended to experience less relationship conflict with that teammate (*b* = –0.83, *SE* = 0.22, *t* = –3.81, *p* < 0.001). Likewise, specific to Hypothesis 3b, target effects of leadership effectiveness negatively correlated with target effects of relationship conflict (*b* = –0.71, *SE* = 0.42, *t* = –1.69, *p* = 0.046).

### How Effective Leadership and Followership Relates to Subsequent Team Conflict

The intraclass correlation coefficient revealed moderate between-team variance for relationship conflict [ICC(1) = 27.8%]. This modest ICC(1) value is aligned with the notion that team conflict may not be uniformly perceived by team members (e.g., Shah et al., 2021). As shown in Table 1, and supporting the criterion validity of the single-item measure of dyadic relationship conflict, this measure was strongly and positively associated with the team-referent measure of relationship conflict (*r* = 0.70, *p* < 0.001). The non-significant association between these measures at the individual-level was expected given that the dyadic relationship conflict measure represents a person’s reputation for relationship conflict within the team, whereas the team-referent measure at the individual-level captures the extent to which a team member deviates from the team average in terms of perceiving conflict.

At the team level, effective leadership and followership were highly correlated (*r* = 0.77, *p* < 0.001), which coheres with theory that leadership and followership are mutually reinforcing processes at the team level of analysis (i.e., higher levels of leadership necessitate higher levels of followership), supporting Hypothesis 1c. Nevertheless, the magnitude of the association creates a multicollinearity issue when assessing residualized associations in a regression model. In our models including both predictors (see Supplementary File), team relationship conflict was negatively predicted by team levels of effective followership and positively predicted by team levels of effective leadership. Given the negative zero-order relations between effective leadership and team relationship conflict (see Table 1), it is evident that effective followership is distorting the relation between effective leadership and team conflict outcomes. Indeed, scholars have emphasized that interpretive difficulties can arise with respect to residualized associations when the predictors share meaningful variance (e.g., Vize et al., 2018). As a result of the multicollinearity between leadership and following ratings, we present the results from the multilevel regression analysis in two separate models.

Tables 2, 3 contain the parameter estimates for how leadership and followership, measured at the midpoint of the team’s lifecycle, relate to relationship conflict measured at the end of the team’s lifecycle. As predicted, latent team scores of effective followership were significantly negatively related to latent team scores of relationship conflict using the dyadic-referent measure (i.e., reflecting the cumulative expression of relationship conflict between specific pairs of teammates) and the team-referent measure (i.e., reflecting the general perception team members have regarding the amount of relationship conflict in the team). The negative relationship between effective leadership at the team level and subsequent relationship conflict was not significant for both the dyadic-referent and team-referent measures.

**TABLE 2:** Followership at midpoint predicting team conflict late in the team life cycle.

	Dyad-referent relationship conflict	Team-referent relationship conflict
**Regression coefficients (fixed effects)**		
Gender (L1)	–0.02 (0.05)	0.02 (0.08)
Work Experience (L1)	–0.14** (0.05)	–0.03 (0.08)
Followership (L1)	–0.44*** (0.09)	0.05 (0.08)
Team Gender (L2)	0.09 (0.12)	0.24 (0.13)
Followership (L2)	–0.27* (0.12)	–0.49*** (0.12)
**Variance components (random effects)**		
L1 residual	0.78*** (0.07)	1.00*** (0.01)
L2 residual	0.92*** (0.07)	0.67*** (0.12)
Intercept	6.48*** (1.30)	8.45*** (1.31)

L1 = individual level; L2 = team level. ***p ≤ 0.001; **p ≤ 0.01; *p ≤ 0.05; All regression coefficients are standardized; ( ) denotes standard error.

*Demographic variables measured in September 2018, several months before the project teams were assembled. Predictor variables were measured in January 2019 and criterion variables were measured in March 2019.*

*Gender at L1 is coded 1 = female and 0 = male; Team gender at L2 is coded 1 = mixed-gender team and 0 = all-male team.*

**TABLE 3:** Leadership at midpoint predicting team conflict late in the team life cycle.

	Dyad-referent relationship conflict	Team-referent relationship conflict
**Regression coefficients (fixed effects)**		
Gender (L1)	–0.02 (0.06)	0.02 (0.08)
Work experience (L1)	–0.14* (0.06)	–0.02 (0.08)
Leadership (L1)	–0.17* (0.08)	–0.02 (0.07)
Team gender (L2)	0.07 (0.13)	0.23 (0.14)
Leadership (L2)	–0.20 (0.16)	–0.30 (0.17)
**Variance components (random effects)**		
L1 residual	0.94*** (0.04)	1.00*** (0.01)
L2 residual	0.95*** (0.06)	0.82*** (0.11)
Intercept	5.70** (1.73)	6.42*** (1.85)

L1 = individual level; L2 = team level. ***p ≤ 0.001; **p ≤ 0.01; *p ≤ 0.05; All regression coefficients are standardized; ( ) denotes standard error.

*Demographic variables measured in September 2018, several months before the project teams were assembled. Predictor variables were measured in January 2019 and criterion variables were measured in March 2019.*

*Gender at L1 is coded 1 = female and 0 = male; Team gender at L2 is coded 1 = mixed-gender team and 0 = all-male team.*

## Discussion

We examined intrateam conflict from the perspective of shared leadership and followership. First, we examined the dyadic relations between leadership and followership to test our assertion that followership is a necessary ingredient for high levels of leadership to exist within a team (H1a–H1c). The results of our bivariate social relations analysis support this assertion, as leadership and followership ratings were tightly linked at the dyadic level. Next, we hypothesized that uniquely rating someone as an effective follower (H2a) or an effective leader (H2b) would decrease the likelihood of experiencing interpersonal conflict with that person at the dyadic level. We also predicted that having a reputation for effective followership (H3a) or effective leadership (H3b) would relate negatively to being viewed as a conflict hub within the team. We found support for both sets of hypotheses, highlighting the importance of both leadership and followership behaviors for managing interpersonal relations between team members. Finally, we hypothesized that team levels of effective followership and leadership would be negatively related to team levels of relationship conflict (H4a and H4b). As predicted, effective followership was negatively related to team levels of conflict (H4a). We did not find support for our hypothesis that effective leadership would predict lower levels of team-referent relationship conflict (H4b). These results suggest that followership represents a key ingredient for buffering against relationship conflict at the team level.

### Theoretical Implications

Shamir (2007) suggested that leadership is “co-produced” by leaders and followers. Our results support the notion of co-production of leadership in small project teams and the reconsideration of the role of followership in shared leadership theory. The dyadic analyses show that team members who have a reputation for effective leadership also tend to have a reputation for effective followership behaviors (evidenced by the strong positive target-target covariance), and teammates who rate someone as a particularly effective leader tend to also rate that teammate as a particularly effective follower (evidenced by the positive intrapersonal relationship covariance). These findings suggest that effective leadership in teams without a formally prescribed hierarchy (i.e., teams with no appointed leader) requires a collaborative and communal orientation. This does not mean, however, that followership on its own is sufficient for achieving desirable team outcomes. Indeed, our results also show that uniquely rating a teammate higher on leadership is associated with being rated higher by that same person on followership (positive interpersonal relationship covariance). Taken together, the results of the bivariate social relations analyses support the notion that acts of leading and following are mutually reinforcing processes that operate in tandem to shape group processes as outlined in the leadership process model (Uhl-Bien et al., 2014). Given that effective followership is rarely measured in conjunction with effective leadership in teams, these results represent an important step forward in shared leadership research by empirically demonstrating the dyadic relation between both leadership and followership in self-managed teams without a formally prescribed hierarchy.

Our results concerning the relations between leadership, followership, and relationship conflict demonstrate that effective followership is a necessary condition for functional team dynamics. The results from our dyadic analyses demonstrate that both effective leadership and followership are necessary in teams, and can reduce the likelihood of engaging in dyadic conflict and becoming a conflict hub within the team. However, when we zoom out to the team level, followership distinguishes itself as a key variable of interest in relation to interpersonal tensions throughout the team. This is evidenced by the significant negative relation between team conflict and effective followership, but not effective leadership, at the team level. These results indicate that higher levels of effective followership throughout the team may be more important than effective leadership with respect to preventing interpersonal conflict on a larger scale. The essential role of followership in predicting team conflict outcomes specifically is likely due to the communal nature of followership. That is, the cooperative and other-focused behaviors involved in followership may act as a balm over interpersonal frictions in a way that effective leadership does not, thereby explaining more unique variance in team conflict outcomes. Overall, our results support the notion that the social coordinating nature of followership (e.g., Van Vugt, 2006) provides the necessary lubricant to allow interdependent teams to navigate logistical challenges.

### Practical Implications

The concept of shared leadership has already made an impact on the business world, with many organizations embracing the notion of distributing the leadership role in teams. However, many leaders find the practice of implementing shared leadership challenging (e.g., Fitzsimons, 2016). This may be due to the fact that leadership is almost exclusively taught as a singular practice in business schools (O’Toole et al., 2003) or because existing power structures in organizations make it difficult to distribute influence and decision-making power (e.g., Toegel and Jonsen, 2016). Regardless of the cause, the enthusiasm expressed regarding shared leadership may soon be dampened by the reality that it is more difficult in practice than in theory. Further, as evidenced by the opening example, having too many team members trying to assert their dominance and influence can cause problems for team performance. As such, it is necessary for the success of future shared leadership initiatives in organizational and other teams to understand the conditions that enable leadership to be shared effectively. The results of this study suggest that one way to increase the success of a shared leadership structure is to encourage leaders in such structures to engage in followership with their peers. Communicating such expectations in advance, particularly when teams are composed of individuals who are used to leading vertically, is likely to make a positive impact on teams adopting flatter structures.

Additionally, the findings from our analyses involving effective followership and team conflict have implications for organizational teams wishing to facilitate positive interpersonal dynamics and reduce relationship conflict. Meta-analytic data have clearly demonstrated the negative impact of interpersonal conflict on team success (e.g., De Dreu and Weingart, 2003). Therefore, understanding the conditions and interpersonal behaviors that buffer against undesirable team conflict states is of great interest to organizational research. Followership at the midpoint was significantly negatively related to subsequent relationship conflict at both the dyadic and team level, which suggests that teams may find it beneficial to focus on establishing and maintaining cooperative and communal orientation as a preventative measure against this form of conflict.

### Limitations and Future Directions

This study explored a novel approach to shared leadership theory by examining the role of effective followership in small project teams. The results of the study show promise for the future of followership research; however, a few limitations should be noted. Although the current study used a prospective design, having three or more timepoints would allow researchers to examine how the leadership and followership roles are shared throughout the team’s lifespan and how changes in levels of followership and leadership connect to conflict states. Future research would benefit from tracking the emergence and implications of these behaviors across time.

We used SRM to examine effective leadership and followership. Although this method allows us to zoom in on interpersonal perceptions of leadership, followership, and conflict, it limits our ability to draw conclusions about the structure of the leader-follower dynamics occurring within the team. Using alternate approaches such as social network analysis would enable insight into the structure of relationship patterns across a group and would be particularly helpful in larger group settings (Carter et al., 2015). Additionally, we examined the distinct behaviors inherent to effective leadership and followership in our study. Although this is consistent with the leadership process model (Uhl-Bien et al., 2014), this approach did not account for the overlapping attributes and behaviors that may be important for both leaders and followers (e.g., competence; team skills). Given the high correlation between leadership and followership at the team level, future research would benefit from teasing out the variance attributed to behaviors that map uniquely onto leadership/followership. Finally, although our project teams were immersed in an ecologically valid context, the nature of our design does not permit us to draw causal conclusions regarding the relationship between leadership, followership, and relationship conflict. Therefore, examining the effect of leader-follower dynamics on relationship conflict in a controlled experimental setting would also be a fruitful pursuit for future research.

## Conclusion

Leadership and followership are distinct, but complementary, patterns of behaviors that work together to facilitate positive team dynamics. Despite theoretical acknowledgment of the necessity of lateral influence in shared leadership models, followership is rarely measured in conjunction with leadership in relation to team functioning. In the current study, we proposed a model that examines leader-follower dynamics as a predictor of relationship conflict at both the dyadic and team level. At the dyadic level, we found that uniquely rating a teammate high on effective followership and leadership was negatively related to experiencing interpersonal conflict with that person. We also found that those with a reputation for effective followership and leadership in their teams were less likely to be viewed as a source of conflict. Interestingly, we found that team level followership, but not team level leadership, was significantly negatively correlated with team relationship conflict. The results of the study indicate that followership is an important predictor of team-level conflict and highlight the interdependent nature of leadership and followership roles.

## Data Availability Statement

The original contributions presented in the study are publicly available. This data can be found here: https://osf.io/rqp27/.

## Ethics Statement

The studies involving human participants were reviewed and approved by The Office of Human Research Ethics, Western University. The patients/participants provided their written informed consent to participate in this study.

## Author Contributions

NB: conceptualization, data cleaning, methodology, and writing – original draft. AB: conceptualization, funding acquisition, methodology, formal analysis, supervision, and writing – review and editing. Both authors contributed to the article and approved the submitted version.

## Conflict of Interest

The authors declare that the research was conducted in the absence of any commercial or financial relationships that could be construed as a potential conflict of interest.

## Publisher’s Note

All claims expressed in this article are solely those of the authors and do not necessarily represent those of their affiliated organizations, or those of the publisher, the editors and the reviewers. Any product that may be evaluated in this article, or claim that may be made by its manufacturer, is not guaranteed or endorsed by the publisher.

## Footnotes

1 These variables represent a subset of measures from a large-scale longitudinal investigation of project design teams across time, which entails personality measures, other emergent team states (e.g., team potency, team satisfaction, team efficacy) and criterion variables of interest (e.g., perceptions of status and inclusion). However, the variables and analyses presented in the current paper do not overlap with these other projects.

2 Target-target covariance is independent of rater bias in the context of SRM due to partitioning target variance from perceiver variance, and a rater positivity bias is reflected in the perceiver-perceiver covariance.

3 Although we did not have predictions for univariate dyadic reciprocity among followership or leadership, it should be noted that uniquely rating a teammate higher in followership was not associated with being rated by that same teammate as higher (or lower) on followership (*b* = 0.27, *SE* = 8.24, *t* = 0.03, *p* = 0.974). However, leadership effectiveness exhibited dyadic reciprocity, such that uniquely rating a teammate higher in leadership was associated with being rated by that same teammate as higher on leadership (*b* = 28.58, *SE* = 8.55, *t* = 3.34, *p* = 0.001).

## References

[B1] Agho A. O. (2009). erspectives of senior-level executives on effective followership and leadership. *J. Leadersh. Organ. Stud.* 16 159–166. 10.1177/1548051809335360

[B2] Aime F. Humphrey S. DeRue D. S. Paul J. B. (2014). The riddle of heterarchy: power transitions in cross-functional teams. *Acad. Manag. J.* 57 327–352. 10.5465/amj.2011.0756

[B3] Avolio B. J. Sivasubramaniam N. Murry W. D. Jung D. Garger J. W. (2003). “Assessing shared leadership: development and preliminary validation of a team multifactor leadership questionnaire,” in *Shared Leadership: Reframing the Hows and Whys of Leadership*, eds Pearce C. L. Conger J. A. (Thousand Oaks, CA: SAGE Publications, Inc), 143–172. 10.4135/9781452229539.n7

[B4] Back M. D. Kenny D. A. (2010). The social relations model: how to understand dyadic processes. *Soc. Pers. Psychol. Compass* 4 855–870. 10.1111/j.1751-9004.2010.00303.x

[B5] Bastardoz N. Van Vugt M. (2019). The nature of followership: evolutionary analysis and review. *Leadersh. Q.* 30 81–95. 10.1016/j.leaqua.2018.09.004

[B6] Behfar K. J. Mannix E. A. Peterson R. S. Trochim W. M. (2011). Conflict in small groups: the meaning and consequences of process conflict. *Small Group Res.* 42 127–176. 10.1177/1046496410389194

[B7] Bendersky C. Hays N. A. (2012). Status conflict in groups. *Organ. Sci.* 23 323–340. 10.1287/orsc.1110.0734 19642375

[B8] Benson A. J. Hardy J. Eys M. (2016). Contextualizing leaders’ interpretations of proactive followership. *J. Organ. Behav.* 37 949–966. 10.1002/job.2077

[B9] Bergman J. Z. Rentsch J. R. Small E. E. Davenport S. W. Bergman S. M. (2012). The shared leadership process in decision-making teams. *J. Soc. Psychol.* 152, 17–42. 10.1080/00224545.2010.538763 22308759

[B10] Carson J. B. Tesluk P. E. Marrone J. A. (2007). Shared leadership in teams: an investigation of antecedent conditions and performance. *Acad. Manag. J.* 50 1217–1234. 10.5465/amj.2007.20159921

[B11] Carter D. R. DeChurch L. A. Braun M. T. Contractor N. S. (2015). Social network approaches to leadership: an integrative conceptual review. *J. Appl. Psychol.* 100 597–622. 10.1037/a0038922 25798551

[B12] Ceri-Booms M. Curşeu P. L. Oerlemans L. A. (2017). Task and person-focused leadership behaviors and team performance: a meta-analysis. *Hum. Resour. Manag. Rev.* 27 178–192. 10.1016/j.hrmr.2016.09.010

[B13] De Dreu C. K. Weingart L. R. (2003). Task versus relationship conflict, team performance, and team member satisfaction: a meta-analysis. *J. Appl. Psychol.* 88 741–749. 10.1037/0021-9010.88.4.741 12940412

[B14] de Jong J. P. Curşeu P. L. Leenders R. T. A. (2014). When do bad apples not spoil the barrel? Negative relationships in teams, team performance, and buffering mechanisms. *J. Appl. Psychol.* 99 514–522. 10.1037/a0036284 24661274

[B15] Doucet O. Poitras J. Chênevert D. (2009). The impacts of leadership on workplace conflicts. *Int. J. Confl. Manag.* 20 340–354. 10.1108/10444060910991057

[B16] Drescher M. A. Korsgaard M. A. Welpe I. M. Picot A. Wigand R. T. (2014). The dynamics of shared leadership: building trust and enhancing performance. *J. Appl. Psychol.* 99 771–783. 10.1037/a0036474 24731178

[B17] Felps W. Mitchell T. R. Byington E. (2006). How, when, and why bad apples spoil the barrel: negative group members and dysfunctional groups. *Res. Organ. Behav.* 27 175–222. 10.1016/S0191-3085(06)27005-9

[B18] Fitzsimons D. (2016). *How Shared Leadership Changes our Relations at Work.* Brighton, MA: Harvard Business Review.

[B19] Goffin R. D. Olson J. M. (2011). Is it all relative? Comparative judgments and the possible improvement of self-ratings and ratings of others. *Perspect. Psychol. Sci.* 6 48–60. 10.1177/1745691610393521 26162115

[B20] Groysberg B. Polzer J. T. Elfenbein H. A. (2011). Too many cooks spoil the broth: how high-status individuals decrease group effectiveness. *Organ. Sci.* 22 722–737. 10.1287/orsc.1100.0547 19642375

[B21] Homans G. C. (1961). *Social Behavior: Its Elementary Forms.* New York: Harcourt Brace & World.

[B22] Humphrey S. E. Aime F. (2014). Team microdynamics: Toward an organizing approach to teamwork. *Acad. Manag. Ann.*, 8, 443–503. 10.5465/19416520.2014.904140

[B23] Humphrey S. E. Aime F. Cushenbery L. Hill A. D. Fairchild J. (2017). Team conflict dynamics: implications of a dyadic view of conflict for team performance. *Organ. Behav. Hum. Decis. Processes* 142 58–70. 10.1016/j.obhdp.2017.08.002

[B24] Hurwitz M. Hurwitz S. (2015). *Leadership is Half the Story: A Fresh Look at Followership, Leadership, and Collaboration.* Toronto, ON: University of Toronto Press.

[B25] Jehn K. Rispens S. Jonsen K. Greer L. (2013). Conflict contagion: a temporal perspective on the development of conflict within teams. *Int. J. Confl. Manag.* 24 352–373. 10.1108/IJCMA-05-2011-0039

[B26] Jehn K. A. (1995). A multimethod examination of the benefits and detriments of intragroup conflict. *Adm. Sci. Q.* 40 256–282.

[B27] Jehn K. A. (1997). A qualitative analysis of conflict types and dimensions in organizational groups. *Adm. Sci. Q.* 42 530–557.

[B28] Katz D. Kahn R. L. (1978). *The Social Psychology of Organizations*, Vol. 2. New York: Wiley, 528.

[B29] Klein K. J. Ziegert J. C. Knight A. P. Xiao Y. (2006). Dynamic delegation: shared, hierarchical, and deindividualized leadership in extreme action teams. *Adm. Sci. Q.* 51 590–621. 10.2189/asqu.51.4.590 21821037

[B30] Locke E. (2003). “Leadership: starting at the top,” in *Shared Leadership: Reframing the Hows and Whys of Leadership*, eds Pearce C. L. Conger J. A. (Thousand Oaks, CA: SAGE Publications, Inc), 271–284. 10.4135/9781452229539.n13

[B31] Lüdtke O. Marsh H. W. Robitzsch A. Trautwein U. Asparouhov T. Muthén B. (2008). The multilevel latent covariate model: a new, more reliable approach to group-level effects in contextual studies. *Psychol. Methods* 13 203–229. 10.1037/a0012869 18778152

[B32] Molm L. D. (1994). Dependence and risk: transforming the structure of social exchange. *Soc. Psychol. Q.* 57 163–176. 10.2307/2786874

[B33] Muthén L. K. Muthén B. O. (2017). *Mplus User’s Guide. Eighth Edition.* Los Angeles, CA: Muthén & Muthén.

[B34] O’Neill T. A. McLarnon M. J. Hoffart G. C. Woodley H. J. Allen N. J. (2018). The structure and function of team conflict state profiles. *J. Manag.* 44 811–836. 10.1177/0149206315581662

[B35] O’Toole J. Galbraith J. Lawler E. E. III (2003). “The promise and pitfalls of shared leadership: when two (or more) heads are better than one,” in *Shared Leadership: Reframing the Hows and whys of Leadership*, eds Pearce C. L. Conger J. A. (Thousand Oaks, CA: SAGE Publications, Inc), 250–267.

[B36] Park S. Mathieu J. E. Grosser T. J. (2020). A network conceptualization of team conflict. *Acad. Manag. Rev.* 45 352–375. 10.5465/amr.2016.0472

[B37] Pearce C. L. Conger J. A. (2003). “All those years ago,” in *Shared Leadership: Reframing the Hows and Whys of Leadership*, eds Pearce C. L. Conger J. A. (Thousand Oaks, CA: SAGE Publications, Inc), 1–18.

[B38] Redhead D. J. Cheng J. T. Driver C. Foulsham T. O’Gorman R. (2019). On the dynamics of social hierarchy: a longitudinal investigation of the rise and fall of prestige, dominance, and social rank in naturalistic task groups. *Evol. Hum. Behav.* 40 222–234. 10.1016/j.evolhumbehav.2018.12.001

[B39] Schönbrodt F. D. Back M. D. Schmukle S. C. (2012). TripleR: an R package for social relations analyses based on round-robin designs. *Behav. Res. Methods* 44 455–470. 10.3758/s13428-011-0150-4 21909865

[B40] Seers A. Keller T. Wilkerson J. (2003). “Can team members share leadership?: Foundations in research and theory,” in *Shared Leadership: Reframing the Hows and Whys of Leadership*, eds Pearce C. L. Conger J. A. (Thousand Oaks, CA: SAGE Publications, Inc), 77–102. 10.4135/9781452229539.n4

[B41] Seibert S. Sparrowe R. Liden R. (2003). “A group exchange structure approach to leadership in groups,” in *Shared Leadership: Reframing the Hows and Whys of Leadership*, eds Pearce C. L. Conger J. A. (Thousand Oaks, CA: SAGE Publications, Inc), 173–192. 10.4135/9781452229539.n8

[B42] Shah P. P. Peterson R. S. Jones S. L. Ferguson A. J. (2021). Things are not always what they seem: the origins and evolution of intragroup conflict. *Adm. Sci. Q.* 66 426–474. 10.1177/0001839220965186

[B43] Shamir B. (2007). “From passive recipients to active co-producers: followers’ roles in the leadership process,” in *.), Follower-Centered Perspectives on Leadership: A Tribute to the Memory of James R. Meindl*, eds Shamir B. Pillai R. Bligh M. Uhl-Bien M. (Charlotte, NC: Information Age Publishers), ix–xxxix.

[B44] Shamir B. (2012). “Leadership research or post-leadership research: advancing leadership theory versus throwing out the baby with the bath water,” in *Advancing Relational Leadership Research: A Dialogue Among Perspectives*, eds Uhl-Bien M. Ospina S. (Charlotte, NC: Information Age Publishing), 477–500.

[B45] Small E. E. Rentsch J. R. (2011). Shared leadership in teams. *J. Pers. Psychol.* 9 203–211. 10.1027/1866-5888/a000017

[B46] Sunnergren T. (2014). *Is the Big Three Model Sustainable for Miami Heat?.* San Francisco, CA: Bleacher Report.

[B47] Swaab R. I. Schaerer M. Anicich E. M. Ronay R. Galinsky A. D. (2014). The too-much-talent effect: team interdependence determines when more talent is too much or not enough. *Psychol. Sci.* 25 1581–1591. 10.1177/0956797614537280 24973135

[B48] Tjosvold D. (2008). The conflict-positive organization: it depends upon us. *J. Organ. Behav.* 29 19–28. 10.1002/job.473

[B49] Toegel G. Jonsen K. (2016). “Shared leadership in a global context: challenges of transferring control to team members,” in *Advances in Global Leadership*, Bingley (Bingley: Emerald Group Publishing Limited). 151–185. 10.1108/S1535-120320160000009006

[B50] Trudel J. Reio T. G. Jr. (2011). Managing workplace incivility: the role of conflict management styles—antecedent or antidote? *Hum. Resour. Dev. Q.* 22 395–423. 10.1002/hrdq.20081

[B51] Uhl-Bien M. Carsten M. (2018). “Reversing the lens in leadership: positioning followership in the leadership construct,” in *Leadership now: Reflections on the Legacy of Boas Shamir*, eds Katz I. Eilam-Shamir G. Kark R. Berson Y. (Bingley: Emerald Publishing Limited), 195–222.

[B52] Uhl-Bien M. Riggio R. E. Lowe K. B. Carsten M. K. (2014). Followership theory: a review and research agenda. *Leadersh. Q.* 25 83–104. 10.1016/j.leaqua.2013.11.007

[B53] Van Vugt M. (2006). Evolutionary origins of leadership and followership. *Pers. Soc. Psychol. Rev.* 10 354–371. 10.1207/s15327957pspr1004_5 17201593

[B54] Vize C. E. Collison K. L. Miller J. D. Lynam D. R. (2018). Examining the effects of controlling for shared variance among the dark triad using meta–analytic structural equation modelling. *Eur. J. Pers.* 32 46–61. 10.1002/per.2137

[B55] Wall V. D. Jr. Nolan L. L. (1986). Perceptions of inequity, satisfaction, and conflict in task-oriented groups. *Hum. Relat.* 39 1033–1051. 10.1177/001872678603901106

[B56] Wang D. Waldman D. A. Zhang Z. (2014). A meta-analysis of shared leadership and team effectiveness. *J. Appl. Psychol.* 99 181–198. 10.1037/a0034531 24188392

[B57] Weber J. M. Moore C. (2014). Squires: key followers and the social facilitation of charismatic leadership. *Organ. Psychol. Rev.* 4 199–227. 10.1177/2041386613498765

[B58] Weingart L. R. Jehn K. A. (2017). “Manage intra-team conflict through collaboration,” in *The Blackwell Handbook of Principles of Organizational Behaviour*, ed. Locke E. A. (Hoboken, NJ: Blackwell Publishing Ltd), 235–247. 10.1002/9781405164047.ch16

[B59] Yang J. Mossholder K. W. (2004). Decoupling task and relationship conflict: the role of intragroup emotional processing. *J. Organ. Behav.* 25 589–605. 10.1002/job.258

[B60] Yukl G. (2012). Effective leadership behavior: what we know and what questions need more attention. *Acad. Manag. Perspect.* 26 66–85. 10.5465/amp.2012.0088

[B61] Zhang X. Cao Q. Tjosvold D. (2011). Linking transformational leadership and team performance: a conflict management approach. *J. Manag. Stud.* 48 1586–1611. 10.1111/j.1467-6486.2010.00974.x

[B62] Zhu J. Liao Z. Yam K. C. Johnson R. E. (2018). Shared leadership: a state-of-the-art review and future research agenda. *J. Organ. Behav.* 39 834–852. 10.1002/job.2296

